# Reference intervals of hematological parameters in the Chilean adult population and the Mapuche ethnic group

**DOI:** 10.1515/almed-2024-0080

**Published:** 2025-01-03

**Authors:** Pablo J. Letelier, Carolina A. Chicahual, Nicolas F. Arroyo, Daniel P. Monsalves, Rodrigo E. Boguen, Neftalí H. Guzmán

**Affiliations:** Precision Health Research Laboratory, Departmento de Procesos Diagnósticos y Evaluación, Facultad de Ciencias de la Salud, 28046 Universidad Católica de Temuco, Temuco, Chile

**Keywords:** ethnicity, full blood count, hematological parameters, outliers, reference intervals

## Abstract

**Objectives:**

Reference intervals (RI) are an essential tool to support clinical decisions. These may have intra- and inter-individual variations associated with genetic differences and environmental factors. Given that Chile is a multiethnic territory, studying these variables is even more relevant. The purpose of this study was to establish RI for various hematological parameters in the Chilean population and the Mapuche ethnic group.

**Methods:**

A sample of 356 adult individuals (aged 18–65 years), of which 146 belonged to the Mapuche ethnic group, was selected using the indirect a posteriori method from the database of the UC Temuco Clinical Laboratory. The analysis was conducted by sex and ethnicity. The Tukey fences method was employed to detect outliers, and the RIs were established through the non-parametric method recommended by the IFCC.

**Results:**

The median age for the overall sample of the general population was 35 years (female) and 36 years (male). Differences (p<0.05) were found by sex in parameters dependent on hemoglobin and platelets counts. In the analysis by ethnicity, the parameters of RBC, HGB and HCT presented significant differences (p<0.0001).

**Conclusions:**

This study shows that hematological RI vary according to sex and ethnicity, which must be considered in a multiethnic population. This understanding enhances our comprehension of the individual characteristics of each person and facilitates more accurate clinical interpretation.

## Introduction

Reference intervals (RI) are the values between the lower and upper reference limits [1] which describe the variation of biological parameters in individuals who have specific characteristics (reference individuals) [2]. They are essential in clinical decision making, supporting diagnosis, monitoring and epidemiological surveillance in different human pathologies. RI for clinical laboratory tests are generally not studied in different subpopulations, nor are they based on specific epidemiological or clinical profiles, which is a drawback for the interpretation of laboratory tests [2], 3]. The hemogram is one of the most frequently-requested laboratory tests, as it is part of the basic studies required for the diagnostic orientation and evaluation of individuals. Its validity has been maintained over time, evolving with the automation of cell counts, and the incorporation of new parameters such as erythrocyte distribution width (RDW) and platelet distribution width (PDW) [4]. The quantitative parameters of the blood count, as well as the study of the erythrocyte indices, mean corpuscular volume (MCV), mean corpuscular hemoglobin (MCH) and mean corpuscular hemoglobin concentration (MCHC) and RDW, support clinical interpretations in various pathologies (polyglobulia, polycythemia, anemia, etc.). Alterations in the differential variables, such as neutropenia are common in patients undergoing chemotherapy, infections and immunological conditions, as well as neutrophilia in response to infectious diseases, inflammatory disorders and hematological malignancies [4], 5]. Additionally, the use of ratios such as the neutrophil-to-lymphocyte ratio (NLR) and the platelet-to-lymphocyte ratio (PLR) has allowed for more clinical information to be covered, as both have been associated with the inflammatory state of the organism [6].

Due to the difficulty in measuring RI, laboratories usually use the figures provided by data sheets from reagent manufacturers or from international literature. The use of external RI can generate challenges in the correct interpretation and management of patients. The evidence demonstrates significant differences in Ris across different ethnicities [7], with variations related to sex, geographic area [8] and age [9]. The Chilean Law 19,253 recognizes nine indigenous peoples [10], equivalent to 12 % of the national population (2,185,732 people), with the Mapuche group being by far the largest, accounting for 79.18 % of all indigenous individuals (approximately 1,745,147). La Araucanía region in southern Chile is one of the seven regions with the largest indigenous populations, comprising 34.35 %, equivalent to 314,174 people [10].

Despite high social heterogeneity, there is little research in Latin America regarding the determination of RIs in local populations [11]. Those studies that do exist are usually limited to a specific characteristic such as height [12]. In Chile, there are only two articles regarding blood RI, one on reticulocytes in a healthy pediatric population [13] and another that analyzed a sample of military personnel exposed to altitude [14]. Given this paucity of information, it is indeed relevant to determine RIs in the population of the La Araucanía region, especially as the health of the Mapuche population presents an epidemiological pattern of prolonged transition, characterized by high levels of infections and chronic degenerative diseases [15]. Thus, the aim of this study was to establish the RI of hematological parameters in the adult Chilean population and the Mapuche ethnic group.

## Materials and methods

### Study design

Non-experimental, retrospective study with univariate and multivariate analysis. The data were obtained from the UC Temuco Clinical Laboratory, located in the city of Temuco (Araucanía Region, Chile). By means of an indirect *a posteriori* method, a sample of 356 adult individuals (50 % male and 50 % female) was selected, which complies with the method currently recommended by the *International Federation of Clinical Chemistry and Laboratory Medicine* (IFCC), where the non-parametric method requires a sufficient number of individuals (≥120). Of this sample, 146 people were of Mapuche ethnicity who were compared with 146 selected non-Mapuche people. The following hematological parameters measured by Sysmex Xs1000i (Sysmex Corporation, Japan) were studied: leukocyte count (WBC), red blood cell count (RGB), hemoglobin concentration (HGB), hematocrit (HCT), mean corpuscular volume (MCV), mean corpuscular hemoglobin (MCH), mean corpuscular hemoglobin concentration (MCHC), RDW and platelet count (PLT). Relative differential counts of neutrophils (NEUT%), lymphocytes (LINF%), monocytes (MONO%), eosinophils (EO%), basophils (BASO%) and the absolute differential counts of neutrophils (NEUT#), lymphocytes (LINF#), monocytes (MONO#), eosinophils (EO#), and basophils (BASO#) were also included. In addition, the NLR index (using the NEUT#/LINF# ratio) and the PLR index (using the PLT#/LINF# ratio) were calculated. The values were expressed according to the international system of measurements for blood counts.

### Inclusion criteria

Members of the adult population between 18 and 65 years old, apparently healthy, who were assisted in the context of community health promotion and prevention campaigns and who met the criteria established by the Clinical Laboratory to avoid pre-analytical errors, were selected. The group was selected using strict criteria, without significant alterations in laboratory tests. We used a previously selected database, showing no biochemical parameter abnormalities, with data already published [16]. The variables considered for the analyses were sex and ethnicity. The criterion used to select ethnicity was that the person chosen had to have at least one indigenous surname [17], duly validated by the National Corporation for Indigenous Development (CONADI) of Chile, and by the electoral service of Chile (Servel).

### Exclusion criteria

Geriatric and pediatric populations were excluded from the study, considering that older individuals or very young people may present physiological variations that are not representative of the typical reference range for a healthy adult population. Data that were found outside the limits obtained in the Tukey fences statistical test were considered as outliers and were excluded from the analysis. Likewise, laboratory parameters that were below or above the clinical decision limits (CDL) were excluded, this helps prevent individuals with undiagnosed underlying conditions from skewing the results, on the other hand, records with missing or inconsistent data were also excluded to avoid errors in the analysis.

### Obtaining a blood sample

The samples were collected in an EDTA-K_3_ tube from individuals who had fasted for between 10 and 12 h, without having performed any mild or strenuous physical activity for at least 8 h previously. Samples were then analyzed within 4 h.

### Statistical analysis

The reference values were obtained using a non-parametric method of the interpercentile interval, recommended by the IFCC [2], and the *Clinical and Laboratory Standards Institute* (CLSI). For each cluster, the mean and standard deviation (SD) values were calculated. The Tukey fences test was used to detect outliers, and for establishing the lower (Q1 – (1.5 × IQR)) and upper (Q3 + (1.5 × IQR)) limits, with IQR being the interquartile range (IQR=Q3 – Q1), then RI calculations were performed using the non-parametric indirect method based on interpercentile ranks, a method that calculates the rank numbers of the 2.5 and 97.5 percentiles as Lower limit=0.025 (n + 1) and Upper limit=0.975 (n + 1), respectively. The confidence interval of each percentile was determined using the binomial distribution. To determine the differences in the sex variable, the Mann-Whitney test was used. A p-value less than 0.05 (p<0.05) was considered to be statistically significant.

### Research ethics

This study was approved by the accredited Research Ethics Committee of the Universidad Católica de Temuco (document number 011601/23) and performed in compliance with the World Medical Association Declaration of Helsinki (ethical principles for medical research involving human subjects).

## Results

This study involved a sample of 356 individuals to obtain RI in the general population in Chile, with a median age of 35 years in women and 36 years in men. To evaluate potential differences associated with ethnicity, data from 146 individuals who met the criteria to be considered of Mapuche ethnicity were used, with a distribution by sex of 73 % women and 27 % men, with a median age of 34 years for the Mapuche and non-Mapuche groups. To establish RI through the non-parametric method, visual inspection was carried out on each parameter through histograms together with the Tukey fences method that allowed outlier values to be excluded. Tables 1 and 2 present the lower and upper limits of the calculated RI, outliers, mean, and SD for the 21 laboratory tests stratified by sex and Mapuche ethnicity. A low data exclusion rate was observed, with a range of 1–24 outliers found in the parameters studied.

**Table 1:** Reference intervals of hematological parameters in the general population by gender (n=356).

Parameter	Units	Gender	RI	Confidence interval (95 %)	Outliers, n	Mean	SD	Difference female to male (p-value)
RBC	×10^12^/L	F	3.84 – 4.85	(3.34–3.91) – (4.76–5.06)	4	4.363	0.6566	<0.0001^a^
		M	4.27 – 5.81	(4.19–4.44) – (5.69–6.12)	23	5.78	1.86	
HCT	L/L	F	33.8 – 40.5	(33–34.6) – (39.9–41.8)	10	37.55	5.108	<0.0001^a^
		M	38.5 – 48.2	(36.1–39.1) – (47.5–49.6)	23	49.43	16.13	
HGB	g/L	F	113 – 137	(110–116) – (135–138)	14	126.7	17.83	<0.0001^a^
		M	131 – 169	(124–133) – (166–173)	23	170.9	55.7	
MCH	pg	F	25.9 – 32.3	(25.3–26.8) – (31.5–33)	13	29.11	4.491	0.3488
		M	26.8 – 32.9	(16.1–27.8) – (31.7–34.2)	24	33.58	10.9	
MCV	fL	F	78.9 – 94.7	(76.4–79.6) – (93.3–97.2)	11	86.23	12.8	0.0086^a^
		M	78.1 – 93.9	(75.8–80) – (92.2–97.4)	23	97.15	31.63	
MCHC	g/L	F	318 – 353	(313–321) – (353–360)	6	339.3	44.7	<0.0001^a^
		M	332 – 362	(324–333) – (360–374)	23	392.2	123.9	
PLT	×10^9^/L	F	160 – 393	(127–188) – (365–425)	9	272.3	69.39	<0.0001^a^
		M	155 – 367	(100–168) – (337–410)	21	275.4	111	
RDW-SD	fL	F	36.1 – 46.4	(35.6–37) – (45.4–49)	3	41.26	2.6	0.10
		M	36.5 – 45.5	(36–36.8) – (44.6–46.7)	23	40.8	2.24	
NLR	n/a	F	0.76 – 3.06	(0.64–0.91) - (2.97–3.37)	10	1.73	0.58	0.0382^a^
		M	0.75 – 2.93	(0.57–0.83) – (2.54–3.34)	9	1.59	0.55	
PLR	n/a	F	66 – 211	(44–73) – (204–229)	6	127	37.5	0.0010^a^
		M	50 – 273	(14–56) – (237 – 314)	4	119	52.6	
WBC	×10^9^/L	F	4.07–10.95	(2.17–4.25) – (10.5–11.63)	4	6.889	2.055	0.0978
		M	4.32 – 11.4	(3.87–4.65) – (10.36–11.97)	15	7.87	3.008	
BASO#	×10^9^/L	F	0 – 0.06	(0–0.01) – (0.05–0.07)	1	0.02	0.014	0.0094^a^
		M	0.01 – 0.07	(0–0.07) – (0.07–0.08)	1	0.031	0.017	
EO#	×10^9^/L	F	0.03 – 0.47	(0–0.05) – (0.04–0.05)	12	0.22	0.25	0.0751
		M	0.05 – 0.57	(0.04–0.06) – (0.49–0.58)	6	0.25	0.16	
NEUT#	×10^9^/L	F	1.77 – 6.68	(1.45–2.021) – (5.95–7.28)	4	3.85	1.32	0.0110^a^
		M	1.99 – 5.81	(1.46–2.15) – (5.24–6.03)	13	3.88	1.58	
LYMPH#	×10^9^/L	F	1.21 – 3.82	(0.96–1.3) – (3.53–4.01)	4	2.21	0.68	0.1421
		M	1.25 – 3.55	(0.92–1.4) – (3.39–4.16)	9	2.46	1.08	
MONO#	×10^9^/L	F	0.27 – 0.84	(0.26–0.31) – (0.81–0.95)	4	0.51	0.15	<0.0001^a^
		M	0.33 – 0.9	(0.29–0.36) – (0.82–0.99)	11	0.62	0.21	
BASO%	Percent	F	0 – 0.9	(0–0.1) – (0.8–1)	5	0.42	0.26	0.0126^a^
		M	0.1 – 1	(0–0.2) – (0.9–1.1)	5	0.47	0.26	
LYMPH%	Percent	F	18.9 – 50.1	(13.4–20.5) – (45–52)	3	32.92	7.79	0.1421
		M	16.8 – 53	(14.3–20.6) – (49.5–56.1)	5	35.44	10.8	
EO%	Percent	F	0.5 – 6.8	(0–0.9) – (6.2–7)	9	3.28	2.16	0.0751
		M	0.6 – 7.9	(0.5–1) – (7.3–9.4)	5	3.59	2.35	
NEUT%	Percent	F	38 – 72.4	(35.7–40.7) – (69.5–73.9)	4	55.68	8.76	0.0081^a^
		M	37.7 – 72	(31.8–39.6) – (67.3–73.8)	7	54.86	12.8	
MONO%	Percent	F	4.5 – 11.2	(3.4–5.2) – (10.9–12.6)	2	7.69	1.79	<0.0001^a^
		M	5.5 – 11.9	(5.3–6.2) – (11.5–12.5)	9	9.0	2.23	

As determined by the Mann-Whitney test for statistical significance. ^a^Values with significant differences between males and females, p<0.05. RI, reference intervals; SD, standard deviation; F, female; M, male; RBC, red blood cells, HCT, hematocrit; HGB, hemoglobin; MCV, mean cell volume; MCH, mean cell hemoglobin; MCHC, mean cell hemoglobin concentration; PLT, platelets; RDW, RBC distribution width; NLR, neutrophil to lymphocyte ratio; n/a, does not apply; PLR, platelet to lymphocyte ratio; WBC, white blood cells; BASO, basophils; EO, eosinophils; NEUT, neutrophils; LYMPH, lymphocytes; MONO, monocytes; %, relative count; #, absolute count.

**Table 2:** Reference intervals of hematological parameters by ethnicity (n=146).

Parameter	Units	Ethnicity	RI	Confidence Interval (95 %)	Outlier	Mean	SD	Difference Mapuche to non-Mapuche (p-value)
RBC	×10^12^/L	MP	3.94 – 5.6	(3.47–4.16) – (5.54–5.83)	20	5.413	2.144	<0.0001^a^
		NMP	3.89 – 5.06	(3.75–3.94) – (4.94–5.27)	1	4.353	0.33	
HCT	L/L	MP	32.6 – 46.9	(29.7–33.8) – (46.9–48.6)	21	45.86	18.29	<0.0001^a^
		NMP	33 – 41.6	(31–34) – (41.3–41.7)	4	36.98	2.553	
HGB	g/L	MP	103 – 165	(98–110) – (160–174)	21	155.4	62.4	<0.0001^a^
		NMP	106 – 146	(103–110) – (143–148)	4	125.6	11.26	
MCH	pg	MP	25.7 – 31.3	(25–26.2) – (31.4–32.3)	24	33.07	12.55	0.2265
		NMP	25.8 – 32.3	(25.8–26.1) – (31.5–33.3)	11	28.92	2.378	
MCV	fL	MP	76.7 – 94.9	(76–7) – (91.3–93.9)	22	97.64	36.99	0.5297
		NMP	78.8 – 94.6	(74.2–78.9) – (92.9–97.4)	7	85.17	5.5	
MCHC	g/L	MP	319 – 358	(308–325) – (355–366)	21	389.4	145	0.5707
		NMP	318 – 358	(315–321) – (354–362)	6	339.2	11.5	
WBC	×10^9^/L	MP	4.06 – 9.48	(3.51–4.34) – (10.21–11.76)	11	7.74	3.48	0.2059
		NMP	4.06 – 10.31	(2.17–4.19) – (9.45–11.1)	2	6.696	1.784	
RDW	fL	MP	35.9 – 47.2	(35.6–37.5) – (44.9–46.1)	19	47.57	18.46	0.9527
		NMP	36.4 – 45.5	(35.6–37) – (45.4–47.2)	3	41.29	2.776	
PLT	×10^9^/L	MP	175 – 458	(160–192) – (425–466)	11	305.3	127.6	0.0615
		NMP	171 – 383	(162–181) – (355–397)	7	265.5	67.48	
NLR	n/a	MP	0.88 – 3.26	(0.6–0.92) – (2.84–3.76)	7	1.77	0.61	0.3520
		NMP	0.69 – 3.41	(0.64–0.8) – (2.78–3.47)	9	1.70	0.62	
PLR	n/a	MP	72 – 224	(64–84) – (209–248)	9	132	39.2	0.1944
		NMP	57 – 229	(44–69) – (211–248)	6	127	44.4	
BASO%	Percent	MP	0 – 1	(0–0.2) – (0.8–1)	9	0.48	0.34	0.2168
		NMP	0 – 0.9	(0–0.9) – (0.8–1)	4	0.4	0.25	
EO%	Percent	MP	0.7 – 6.6	(0.1–0.9) – (5.4–7.1)	12	3.48	3.06	0.7048
		NMP	0.5 – 6.9	(0–0.8) – (6.1–7)	7	3.21	2.3	
NEUT%	Percent	MP	39.9 – 77.5	(32.2–41.4) – (69.3–78.8)	7	59.38	19.25	0.2772
		NMP	37.4 – 71.8	(36–39.7) – (69.7–73.9)	3	55.15	9.48	
LINF%	Percent	MP	16 – 50.3	(11.9–18.9) – (46.6–53.6)	4	34.41	12.39	0.8114
		NMP	16.3 – 50.1	(13.4–20) – (49.5–54)	3	33.17	8.83	
MONO%	Percent	MP	4.2 – 11.5	(3.8–5.1) – (10.4–12.4)	10	8.42	11.96	0.0735
		NMP	5.2 – 12	(3–5.3) – (11.2–13)	0	8.07	1.93	
BASO#	×10^9^/L	MP	0 – 0.06	(0–0.1) – (0.06–0.07)	8	0.032	0.023	0.2131
		NMP	0 – 1	(0–1) – (1–2)	0	0.025	0.014	
LINF#	×10^9^/L	MP	0.03 – 0.45	(0.01–0.06) – (0.37–0.46)	13	0.23	0.21	0.8772
		NMP	0.03 – 0.5	(0–0.05) – (0.43–0.5)	9	0.21	0.2	
EO#	×10^9^/L	MP	1.7 – 6.78	(1.32–1.97) – (6.18–6.78)	7	4.09	1.94	0.1738
		NMP	1.8 – 6.02	(1.46–2.01) – (5.68–6.59)	7	3.73	1.4	
NEUT#	×10^9^/L	MP	1.09 – 3.41	(0.8–1.34) – (2.95–3.43)	8	2.27	0.95	0.7108
		NMP	1.04 – 3.35	(0.29–1.21) – (0.29–4.01)	3	2.18	0.71	
MONO#	×10^9^/L	MP	0.26 – 0.86	(0.2–0.32) – (0.76–0.87)	8	0.57	0.31	0.2181
		NMP	0.31 – 0.95	(0.27–0.32) – (0.81–10.95)	2	0.53	0.16	

As determined by the Mann-Whitney test for statistical significance. ^a^Values with significant differences between males and females, p <0.05. RI, reference intervals; SD, standard deviation; F, female; M, male; RBC, red blood cells; HCT, hematocrit; HGB, hemoglobin; MCV, mean cell volume; MCH, mean cell hemoglobin; MCHC, mean cell hemoglobin concentration; PLT, platelets; RDW, RBC distribution width; NLR, neutrophil to lymphocyte ratio; n/a, does not apply; PLR, platelet to lymphocyte ratio; WBC, white blood cells; BASO, basophils; EO, eosinophils; NEUT, neutrophils; LYMPH, lymphocytes; MONO, monocytes; %, relative count; #, absolute count; MP, Mapuche population; NMP, non-Mapuche population.

The RI obtained for the general adult population, separated by sex (men and women), are presented in Table 1 and the analysis according to ethnicity (Mapuche and non-Mapuche) in Table 2. Given that the sample size for individuals of Mapuche ethnicity was 146, it was not possible to segregate the analysis by sex, since the minimum number of data points of 120 for each group (men and women) recommended by the IFCC was not met.

It is observed that the RI according to sex, in most of the parameters evaluated, are statistically different in the cases of RBC, HCT, HGB, MCV, MCHC, PLT, NLR and PLR, but not for MCH, WBC, EO%, LINF%, EO# and LINF#. In general, blood count values are higher in men than in women except for MCV, PLT, WBC and absolute neutrophil value (NEUT#). The values of the parameters of the leukocyte differential formula, whether absolute or relative, are very similar in the groups studied.

**Figure 1:**
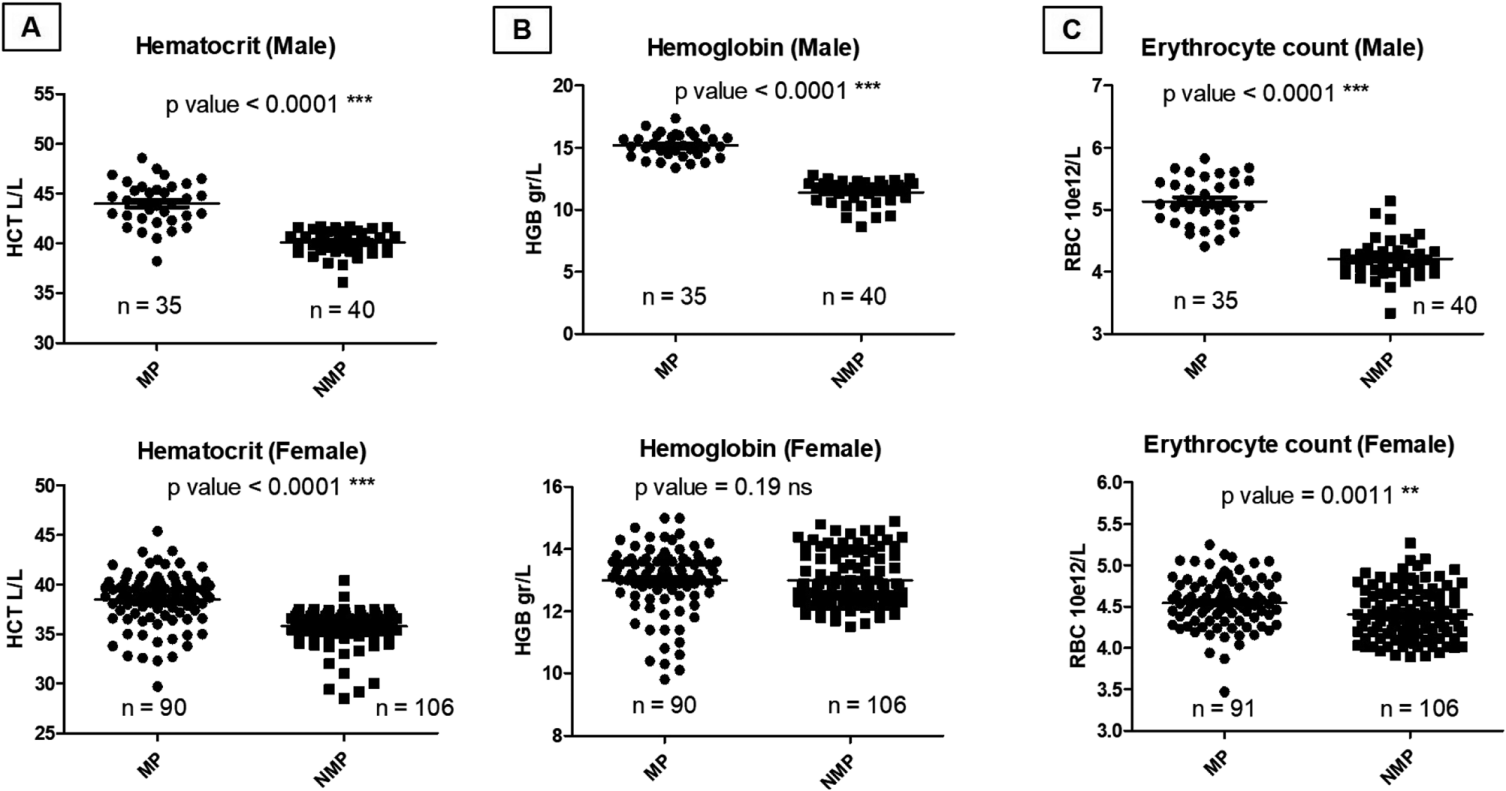
Frequency distribution and statistical significance according to ethnic origin and sex for the HCT, HGB and RBC parameters. (A) Hematocrit (male and female); (B) hemoglobin (male and female); (C) erythrocyte count (male and female), MP, Mapuche population; NMP, non-Mapuche population.

The RI obtained for individuals of Mapuche ethnicity when compared with non-Mapuches, showed significant differences for RBC, HGB and HCT (p<0.0001); it is observed that the mean of the values tends to be generally higher in the Mapuche population for most of the parameters studied. To verify that these results were not associated with the size of the groups by sex, because 72 % were women and 28 % were men for the Mapuche group and 73 % were women and 27 % were men for the non-Mapuche group, these 3 parameters, disaggregated by sex, were analyzed (Figure 1). The HCT was significantly different in women (90 Mapuche and 106 non-Mapuche) with a p-value of <0.0001 (Figure 1), which corroborates the significant difference observed that was not influenced by the size of the groups between men and women. In the analysis of men (Mapuche vs. non-Mapuche; n=35 and n=40 respectively), a statistically significant difference was also observed (p<0.001, Figure 1), with a trend towards higher HCT values in Mapuche men, concordant with RBC values (in both sexes; Mapuche vs. non-Mapuche) and for hemoglobin in men, but not for hemoglobin in women (p-value > 0.05; Figure 1).

Although PLT values were higher at their upper limit in the Mapuche vs. non-Mapuche group (458 × 10^9^/L vs. 383 × 10^9^/L respectively, Table 2), this difference did not reach statistical significance (p-value = 0.0615).

When comparing the RI used by the UC Temuco Clinical Laboratory (data not shown), a greater similarity was observed with those obtained in non-Mapuche individuals, especially in RBC (4.4–5.6 × 10^12^/L); HCT (37.5–47.5 L/L); HGB (125–158 g/L) and PLT (150–450 × 10^9^/L), which shows that this difference is not only observed in this ethnicity, but also in other populations that are studied by reagent manufacturers.

## Discussion

In Latin America, evidence from studies that have established RI in the area of hematology is limited. Chile is no exception in that only two papers have been published on this topic. The first analyzed a random sample of male military volunteers exposed to high altitude in Putre, Chile (3,550 m) [14] whilst the other evaluated reticulocytes in the pediatric population [13]. The RIs obtained from the general population in this study (Table 1) indicated that the vast majority of differences were associated with the sex variable. Women exhibited higher mean corpuscular volume (MCV), platelet counts, and neutrophil-to-lymphocyte ratios (NLR) compared to men. Conversely, hemoglobin concentration, red blood cell (RBC) counts, hematocrit (HCT), and platelet-to-lymphocyte ratios (PLR, upper limit) were higher in the male population. This highlights the importance of sex segregation when calculating RIs in blood counts. Furthermore, when analyzing these results in conjunction with those of other authors, we observe that the RIs obtained here for RBC, MCV, MCH and MCHC are similar to those reported by Rosenfeld et al. [18], Fernández et al. [19] and Adeli et al. [20], although in the latter study, it should be noted that different groups and age ranges were used. Also in these studies, higher RBC values (upper limit, females) are observed compared to our work. Likewise, the RI in the parameters of RBC, HGB and HCT were higher in men than in women, which coincides with studies carried out in Ireland [21], Belgium [22], Spain [23] and Turkey [24]. Lower hemoglobin levels in women is also consistent with the findings of other studies [25]. The MCV and MCH parameters did not present differences according to sex, which is also consistent with the aforementioned studies. Regarding the platelet count, in general there is similarity between studies, except with the work of Rosenfeld et al. [18] where the values in women were lower. Additionally, HCT (for both sexes) and HGB (in women) were higher, while white blood cell (WBC) counts were lower compared to our study [18]. For their part, Sáenz et al. [26], who analyzed an Ecuadorian high Andean population, reported a WBC range of 6.7–7 × 10^9^/L, significantly below the levels found in our study; however the altitudinal conditions of individuals in both studies were notably different. The relatively high WBC count in our study, compared to others, may be associated with factors such as race, circadian variations, pregnancy, stress, exercise, and/or medication use [27], 28]. These differences were particularly evident when compared to studies by Islam et al. [21], Florin et al. [22] and Arbiol et al. [23]. Regarding the leukocyte data (Table 1), minimal differences are observed compared to data reported by the UC Temuco Clinical Laboratory.

Variations in RI by sex may be associated with differences in the endocrine system. Testosterone has been reported to induce erythropoiesis by increasing erythropoietin and suppressing hepcidin [29]. Hepcidin is a peptide that acts by blocking cellular iron flow. Furthermore, estrogen has been shown to directly affect the transcription factor GATA1, leading to a significant increase in the apoptosis of erythroid cells [30]. In addition, fluctuations in sexual steroid hormones during the menstrual cycle result in changes in hematological parameters such as HCT, HGB, neutrophil count and eosinophil count [31]. These modifications stimulate the bone marrow, inducing blood flow of immature erythrocytes into peripheral blood. Combined with the relatively high prevalence of iron deficiency anemia in women during menstruation, these factors explain the differences in RIs in these parameters by sex [32]. Conversely, women’s muscle mass is generally 25–40 % less than that of men [33], resulting in a greater demand for oxygen and blood flow in the latter. Consequently, a higher body mass index is associated with elevated values of WBC, RBC, HGB, HCT and PLT in children and adolescents [34]. Other factors must also be considered, such as strenuous exercise, which can increase the concentration of HGB, HCT and RBC, while potentially decreasing total blood volume (VS) [35]; however, this factor was considered in the exclusion and inclusion criteria of our study.

Regarding RDW, no significant differences were noted between sex and ethnicity. The RI obtained was very similar to that currently used by the UC Temuco Clinical Laboratory (RDW-SD 37–46 fL); however, we could not compare this parameter with those reported by other authors, as we only had RDW expressed as standard deviation (RDW-SD). The primary clinical application of RDW, along with other parameters such as MCV, has been in the study of iron deficiency anemia and β-thalassemia, as well as other nutritional deficiencies causing megaloblastic anemia such as folic acid or vitamin B12 [36]. Nevertheless, different studies have demonstrated that RDW can also be altered in individuals with various cardiovascular pathologies, diabetes, kidney diseases, critically ill patients [37].

Additionally, we calculated the neutrophil-to-lymphocyte ratio (NLR) and platelet-to-lymphocyte ratio (PLR), analyses that reveal the inflammatory state of the subject [6] as they are associated with various processes such as post-thrombolytic side effects in patients with acute ischemic stroke [38]. Currently, very few studies that have determined the RI of these ratios. For example, in the Chinese population, the RI of NLR was found to be 0.43–2.75 for men and 0.37–2.87 for women, while PLR was 36.63–149.13 for men and 43.36–172.68 for women; age-related data was also collected [39]. In South Korea, the RI of NLR was similar to that found in this study, with a mean of 1.7 for men (age range 20–49 years) and a mean of 1.6 for women. Greater similarity in PLR was observed only in the age range over 70 years [40]. In the Iranian population, means of NLR and PLR of 1.70 ± 0.70 and 117.05 ± 47.73, respectively [41]. In the Nigerian population, values of 2.8 (1.2–4.4) for NLR, and 137 (75–199) for PLR [42] were observed, the latter being higher than those found in this study.

Although the concept and usefulness of RI is simple, their establishment is complex as there are factors that depend on the characteristics of the individuals, the inclusion and exclusion criteria, and the equipment and methodologies used in their calculation [24]. This is why their determination requires that pre-analytical and analytical processes be rigorously standardized, with appropriate statistical methods and representative samples of individuals. Some of the studies have limited sample sizes, such as those of Arbiol et al. [23] with 213 data points, Islam et al. with 132 [21], Molina et al. with 135 [28], and Fernández et al. with 250 [19]. However, it should be noted that all the aforementioned studies still examined a greater number of samples than that recommended by the IFCC for non-parametric analysis (120 data points). Conversely, some studies utilized significantly larger sample sizes, such as Rosenfeld et al. with 8,952 [18] and Hollowell et al. with 26,372 [43]. Regarding analytical differences, several types of equipment are used, with most authors preferring the Sysmex XE-2100 hematology counter [22], 23], 26], 28] and the ADVIA 2120i [21]. Likewise, for statistical analysis, some researchers have also used the Tukey fences as a method of excluding outliers [22], 23], whilst others calculate RI from the X±2SD (mean ± 2 standard deviation), which decreases the precision of the intervals. In our case, the interpercentile interval method was used, as recommended by the IFCC [2].

Normally, RIs have been determined in predominantly Caucasian individuals [44], so it is relevant to highlight that Latin America is a multiethnic and multicultural region [45]. Using mitochondrial genome markers, population genetics studies show that the Chilean population has a mainly Amerindian genetic background [46]. Moreover, migratory factors are increase in Chile and also the visibility of Indigenous Peoples in Chilean cities has continued to increase in recent decades [47], mainly triggered by “pull factors”, such as better access to goods and services, development and extraction of natural resources and territorial imbalances. This is why we studied the Mapuche ethnicity variable, and found that the parameters of RBC, HGB and HCT had significant differences (p<0.0001) compared to the non-Mapuche group. Although other parameters were not statistically different, it was observed that the mean values tend to be higher in the Mapuche population in the vast majority of the parameters studied.

Other studies in different ethnicities (Caucasian, Negroid and Asian) have found important differences, such as the RI in Asians having significantly lower estimates of HCT, HGB, HCM, MCHC and mean platelet volume [48]. Black individuals have significantly lower RI for HCT, HGB, HCM, MCHC, total cholesterol, triglycerides and leukocytes, compared to white individuals. Lim et al. [48] observed that Hispanics have lower RI in HCM and MCHC [48]. In the study by Cheng et al. [49] found that the percentages of mononuclear cells and lymphocytes in the Negroid population were higher, and there were decreases in the percentage of granulocytes for all ages and sexes. HCT, MCHC, MCH, and HGB were also lower in all age and sex categories [49].

Regarding the limitations of the study, the restricted size of the sample used in the case of the population of the Mapuche ethnic group did not allow for the establishment of an analysis segregated by sex. Moreover, the inclusion criteria for this ethnic group considered the presence of at least one Mapuche surname, so this analysis probably included individuals of mestizo ancestry. In addition, according to the migration patterns within Chile, which often lead to changes in lifestyles, future studies should focus on studying RI directly in ethnic communities in this, and other regions.

In conclusion, the reference intervals have typically been established predominantly in Caucasian populations, making it important to emphasize that Latin America is a multiethnic and multicultural region. The present study is the first to establish RI for hematological parameters in the adult population and the Mapuche ethnic group in Chile. Some of the parameters present greater precision and variability depending on the population analyzed, its geographical location and sex, whilst others vary according to ethnicity. There are important differences in certain parameters, such as RBC, HCT, and HGB, between the Mapuche population and those used in the UC Temuco clinical laboratory. Therefore, it is important to highlight that adjusting reference intervals (RI) has direct implications for the quality of medical care, supporting more accurate diagnoses and reducing the risk of misinterpretations, such as underdiagnosing anemia or overestimating hematological issues. Additionally, it allows for better treatment adaptation, as reference thresholds reflect the physiological characteristics of each group. In the context of indigenous populations or different ethnic groups, using RIs based on Caucasian data may lead to suboptimal treatments. This also impacts public health policy, enabling more accurate population health assessments and better planning of interventions. This is particularly relevant in regions like Latin America, where ethnic differences must be considered to avoid healthcare inequities. Additionally, it highlights the importance of planning validation studies for proper transferability and potential multicenter studies that account for these variables in particular populations. Finally, these data provide relevant guidance for other studies and clinical practice in our geographic area.
